# Applications of machine learning in tumor-associated macrophages

**DOI:** 10.3389/fimmu.2022.985863

**Published:** 2022-09-23

**Authors:** Zhen Li, Qijun Yu, Qingyuan Zhu, Xiaojing Yang, Zhaobin Li, Jie Fu

**Affiliations:** ^1^ Radiation Oncology Department, Shanghai Jiao Tong University Affiliated Sixth People's Hospital, Shanghai, China; ^2^ Department of Pulmonary and Critical Care Medicine, Ruijin Hospital, Shanghai Jiao Tong University, Shanghai, China; ^3^ Institute of Respiratory Diseases, School of Medicine, Shanghai Jiao Tong University, Shanghai, China

**Keywords:** machine learning, tumor microenvironment, tumor-associated macrophages (TAMs), deep learning, artificial intelligence

## Abstract

Evaluation of tumor-host interaction and intratumoral heterogeneity in the tumor microenvironment (TME) is gaining increasing attention in modern cancer therapies because it can reveal unique information about the tumor status. As tumor-associated macrophages (TAMs) are the major immune cells infiltrating in TME, a better understanding of TAMs could help us further elucidate the cellular and molecular mechanisms responsible for cancer development. However, the high-dimensional and heterogeneous data in biology limit the extensive integrative analysis of cancer research. Machine learning algorithms are particularly suitable for oncology data analysis due to their flexibility and scalability to analyze diverse data types and strong computation power to learn underlying patterns from massive data sets. With the application of machine learning in analyzing TME, especially TAM’s traceable status, we could better understand the role of TAMs in tumor biology. Furthermore, we envision that the promotion of machine learning in this field could revolutionize tumor diagnosis, treatment stratification, and survival predictions in cancer research. In this article, we described key terms and concepts of machine learning, reviewed the applications of common methods in TAMs, and highlighted the challenges and future direction for TAMs in machine learning.

## 1 Introduction

The tumor microenvironment (TME) is a complex system consisting of various components that would shape tumorigenesis, progression and metastasis. In addition to cancer cells, numerous innate immune cells reside within the TME, for instance, macrophages, dendritic cells, neutrophils, myeloid-derived suppressor cells, etc. In the complex environment, tumor-associated macrophages (TAMs), the major immune cells infiltrating tumors, can orchestrate various aspects of tumor biology, such as tumor initiation, progression, metastasis, and even anti-tumor immunosuppression. As crucial drivers in fostering tumor progression, TAMs are standing out as promising targets for diagnosis and new treatments in malignant tumors.

Machine Learning (ML) is a group of data-analytical methods to build predictive models by summarizing past empirical or theoretical literature. Deep learning (DL) is considered an evolution of machine learning. It uses a programmable artificial neural network (ANN) which is inspired by a biological nervous system to make accurate decisions. Recently, ML, DL, in particular, has exhibited a remarkable development with the support of the rapid increase in the storage capacity and processing power of computers. In the era of big data, ML methods have come to attention as their extraordinary ability to process large and heterogeneous data sets in complex biological systems. As P4 (Predictive, Preventive, Personalized, and Participatory) and precision medicine are emerging and gaining traction (1), ML has become integral to modern biological research for its ability to solve challenges not well addressed by traditional methods. There have been many applications of ML in medical research ranging from cancer classification, subtyping, new biomarker discovery, and drug discovery (2–5). Considering the crucial role of TAMs in TME and tumor biology, ML has been widely employed in TAMs-related studies and has achieved successful outcomes.

This review is intended for readers with little knowledge of ML algorithms. Firstly, we briefly review the origins, types, and functions of TAMs. Secondly, we introduce the basic principles and key concepts needed to understand how ML methods could be applied and utilized in cancer research. Thirdly, we discuss the methods and applications at the intersection of ML and TME, especially TAMs. In the end, we highlight the current challenges in ML that need to be addressed, as well as the future directions that could be used to fully realize the potential applications in cancer therapy.

## 2 Origins and types of TAMs

TAMs comprise almost 50% of immune cells infiltrating tumors. They are highly heterogeneous cells that can be divided into two main origins: bone-marrow-derived macrophages (BMDMs) developing from hematopoietic stem cells and tissue-resident macrophages (TRMs) from progenitors seeded into tissues during embryonic development. For a long time, BMDMs have been considered the main effectors in TAMs, but nowadays, TRMs have emerged as an inseparable and essential component in TME (6).

In a simplified view, there are two distinct populations of polarized macrophages, the classical M1 [upon lipopolysaccharide (LPS) and IFNG stimulation] and the alternative M2 (upon IL4 or IL13 stimulation) phenotypes macrophages. Macrophages undergo polarization and get activated in multiple processes during physiological and disease processes (7, 8). M1 and M2 macrophages have different markers, including CD surface receptors, cytokines, chemokines, transcription profiles, etc. (**Table 1**). We have listed the characterized biomarkers, CDs, and cytokines for TAMs identification. M2 macrophages can be further classified into different subtypes, namely M2a (mediated by IL4 and IL13), M2b (mediated by immune complexes (IC) with LPS or IL1R ligand), M2c (mediated by TGFB1, IL10, and glucocorticoids), and M2d (activated by tumor-associated factors, the major part of TAMs) (44, 45). In contrast to proinflammatory, antibacterial, and anti-angiogenic M1 macrophages, M2 macrophages suppress inflammation, facilitate tissue repair, remodeling, angiogenesis, and retain homeostasis under physiological conditions (46, 47).

**Table 1:** M1 and M2 macrophages markers.

Characteristics	M1 (classical)	Reference	M2(alternative)	Reference
Stimuli	LPS/IFNG/CSF2	(9, 10)	IL4/IL13/CSF1	(10, 11)
CDs and MHC	CD68, CD80, CD86, MHC-II	(12–14)	CD68, CD204, CD163, CD206	(15)
Cytokines and Chemokines	IL1B, IL6, IL12, TNF, IFNG CXCL9, CXCL10, CXCL11, CXCL16	(9, 11) (9, 16),	IL10, VEGFA/C, TGFB1 CCL17, CCL18, CCL22, CCL24	(9, 15)
Non-coding RNAs	miR-125b-2	(17)	miR-375	(18)
	miR-16	(19)	miR-34a	(20)
	miR-9	(21)	miR-301a	(22)
	lncRNA-PVT1	(23)	miR-934	(24)
	lncRNA-MEG8	(25)	miR-940	(26)
	lncRNA-GAS5	(27)	let-7b	(28)
	miR-155	(29, 30)	let-7c	(31)
	miR-142-3p	(32)	let-7d-5p	(33)
	miR-146a	(14)	miR-19b-3p	(34)
			lncRNA-MM2P	(35)
Others	NOS2, ROS, HMGB1	(11, 14, 36–38),	PD-1/PD-L1, MMP1/2/9, Arg1, Chil3, Retnla	(39–43)

In general, TAMs contain M2 and small populations of M1 cells (48). However, the distinction between the M1 and M2 states is less clear in TME since TAMs probably display phenotypes anywhere in between these two extremes. Moreover, the phenotype of TAMs dynamically changes with the development and progression of tumors. Each macrophage in TME might show anti- or pro-tumorigenic properties to form a plastic and heterogeneous tumor-promoting totality in response to diverse microenvironmental signals (a mixed M1–M2 phenotype). In a word, the M1 or M2 only phenotype is too simple to elucidate the intricate roles of TAMs in the TME (49–53).

## 3 Roles of TAMs in tumor

**Figure 1:**
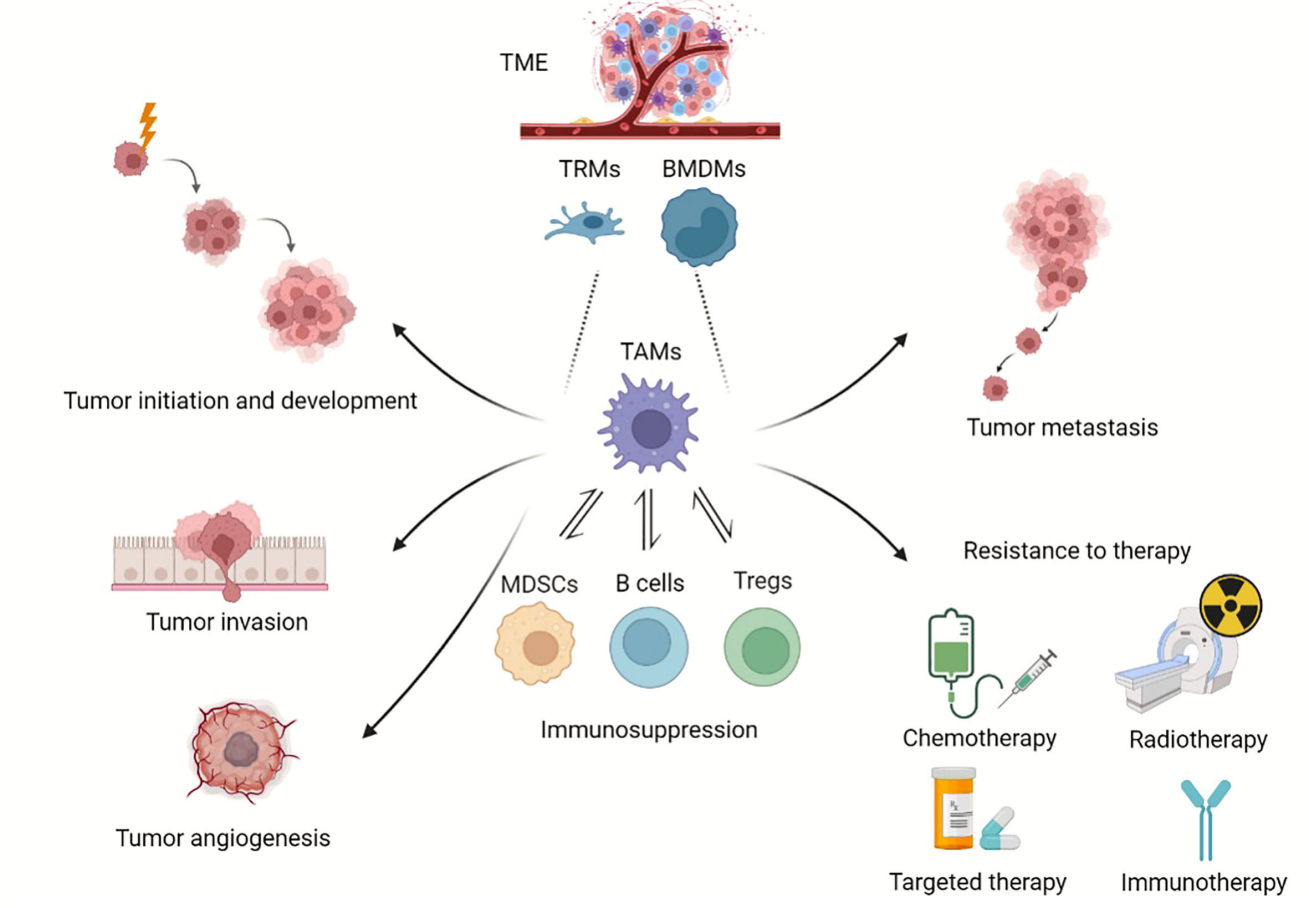
Roles of TAMs in tumor progression. Overview of TAMs in tumor progression. TAMs can derive from BMDMs and TRMs. TAMs provide a niche for tumor initiation and development, participate in angiogenesis, promote tumor metastasis, and enhance resistance to chemotherapy, radiotherapy and immunotherapy. (Created with BioRender.com).

Macrophages are considered essential components in immune defense and immune sentinels combating tumor growth; however, accumulated evidence supports a new tumor-promoting role of macrophages as well. Different from the basic functions of phagocytizing pathogens and apoptotic cell debris, TAMs are equipped to execute a broad repertoire of pro-tumorigenic functions as heterogeneous effectors (**Figure 1**).

### 3.1 TAMs in tumor initiation and development

TAMs profusely infiltrate TME with the ability to suppress anti-tumoral immune surveillance. Accumulating evidence has suggested that TAMs can express a variety of immunosuppressive chemokines and factors which promote tumor cell proliferation and survival, including platelet-derived growth factor (PDGF), epithelial growth factor (EGF), and transforming growth factor beta 1 (TGFB1) (54, 55). The abovementioned chemokines and factors lead to immune cell–cell interactions as well. For instance, TAMs can inhibit anti-tumor immunity by restraining antigen presentation and blocking T cells function, in which case T cells lose their capacity in recognizing and even killing tumor cells (45). Usually, activated cytotoxic T lymphocytes (CTLs) can attack cancer cells to suppress tumor growth, while TAMs express immunosuppressive cytokines, chemokines, and growth factors like IL10 and TGFB1 to make CTLs hyporesponsive (6). As a distinct T-cell subpopulation, regulatory T cells (Tregs) are actively engaged in the maintenance of immunological self-tolerance (56). IL10 and TGFB1 from TAMs can also induce Tregs-mediated immunosuppression (57). Besides, TAMs are able to recruit Tregs *via* CCL22 production, which further suppresses the antitumor immune response of T-cells and fosters tumor growth (58). Moreover, it is worth noting that cancer cells can strongly induce TAMs into pro-tumorigenic phenotype by secreting colony-stimulated factor 1, mucins and exosomes (59–61). To sum up, all these factors work together and make the TME a hospitable site.

### 3.2 TAMs in tumor angiogenesis

Angiogenesis can be briefly defined as the formation of new capillaries from pre-existing blood vessels. It is generally accepted that tumor growth largely depends on angiogenesis since new vessels can supply fresh oxygen and nutrients as well as remove wastes and metabolites. Furthermore, angiogenesis is a vital event in hematogenous metastasis (62). Angiogenesis is activated when pro-angiogenic factors predominate over anti-angiogenic factors (63). As shown in **Table 1**, TAMs can produce diverse pro-angiogenic molecules (VEGF family, PDFG, TGFB1, etc.) and matrix metalloproteinases (MMP) to facilitate angiogenesis. In particular, developing tumors consume oxygen supply rapidly and tend to create an oxygen deficiency condition (hypoxia). It has been increasingly recognized that TAMs massively infiltrate hypoxic regions in tumors and hypoxic macrophages achieve a pro-angiogenic response by directly upregulating the abovementioned pro-angiogenic molecules through hypoxia-inducible factor-1 alpha (HIF1A) (64–67).

### 3.3 TAMs in tumor metastasis

TAMs demonstrate lots of essential functions in tumor biology. In tumor metastasis, it is still a puzzling question how TAMs facilitate tumor spread specifically, though TAMs get involved in almost every process of metastasis. Herein, we provide a quick summary of the fundamental mechanics. First, TAMs within the TME can enhance tumor cell migration and invasion, thereby enabling the escape of tumor cell from the confines of the basement membrane into the surrounding tissues. Second, TAMs are associated with tumor angiogenesis, which, as was previously mentioned, results in tumor intravasation and vasculature-based tumor spread (68). Third, in the immunosuppressive TME, cancer cells can escape from being killed by T cells and prolong cell survival, which make it easier to spread to farther tissues and organs (69). It should be highlighted that tumor metastasis is a process that starts at a very early stage rather than a late event initiated and shaped in advanced cancers. Distant organs are conducive to the survival and outgrowth of primary cancer cells before their arrival. Those ‘primed’ sites are known as ‘pre-metastatic niches’ (PMNs) (70) and special attention has been given to the key role of TAMs in PMNs from clinical evidence (71). Upon the induction of many tumor-secreted factors, TAMs are recruited into the blood and then gather at the pre-metastatic sites (70, 72–74). Meanwhile, TRMs stemming from yolk sac progenitors, like cerebral microglia, liver Kupffer cells, pulmonary alveolar macrophages, and osteoclasts, have been resident in the distant sites before tumorigenesis and get involved in orchestrating PMNs formation following diverse stimulation as well. These macrophages guide circulating tumor cells (CTCs) into the PMNs through enhancing the expression of chemokines and remodeling the extracellular matrix (ECM) into more tumor-favorable structures (75).

### 3.4 TAMs enhance resistance to chemotherapy, radiotherapy and immunotherapy

Emerging cancer research depicts that a high proportion of TAMs infiltration in tumor samples is often associated with shortened survival and poor prognosis in many tumors (76–79). Furthermore, TAMs infiltration is thought to offset therapeutic response to radiotherapy, chemotherapy and targeted therapy, even leading to treatment failure (80, 81). Regarding underlying mechanisms, TAMs can reduce the efficacy of radiotherapy by triggering the anti-apoptotic programs in cancer cells that are resistant to radiotherapy. They also secrete a variety of cytokines and survival factors to mediate the resistance of the solid tumor to many chemotherapy drugs, including IL6 and milk-fat globule-epidermal growth factor-VIII (82, 83). Programmed death ligand 1 (PD-L1), which is thought to be carried by TAMs and is upregulated in response to stimulation of TME-derived factors, has been linked to immune exhaustion *via* the checkpoint ligand/receptor interaction. However, existing studies do not depict a comprehensive picture since another study comes to a contrary conclusion that PD-L1 expression on TAMs, instead of cancer cells, is positively associated with patients’ overall survival (84). Thus, further studies addressing the precise mechanisms involved are urgently needed.

Considering all these functions of TAMs, it is essential to comprehend heterogeneous TAMs and their roles in tumor biology to create and enhance more potent treatments. To date, various molecular strategies against TAMs are currently in preclinical or clinical trials, trying to overcome the knotty problem of immune suppression, such as TAMs recruitment, TAMs depletion and TAMs reprogramming (85).

## 4 Basics of machine learning

The term machine learning was first coined in the 1950s by Arthur Samuel, a computer scientist at IBM (86). Since then, ML has evolved considerably and now is playing a critical role in modern medical science. ML is a subdivision of artificial intelligence and can be briefly defined as enabling algorithms to make accurate predictions based on prior experiences (87). The boundary between conventional statistical techniques and ML is obscure, whilst some terms in ML have similar functions to statistical methods. Some conventional statistical techniques, such as ridge regression can be combined with ML algorithms for prediction (88). One key distinction between ML and traditional statistical methods is that conventional statistic methods focus on the relationship between variables (89). However, ML contributes to identifying patterns from massive data and then performing predictions. Moreover, ML aims to solve more complicated problems, often dealing with high dimensional variables with the technique of feature selection, pattern analysis and dimensionality reduction. As a result, it extends and supplements existing statistical methods by offering tools and algorithms to decipher patterns in enormous, intricate and heterogeneous data sets. Common terminologies and explanations in ML can be seen in **Table 2**.

**Table 2:** Common terminologies and explanations in ML.

Artificial Intelligence	Artificial intelligence is the capability of a computer to perform tasks that are generally completed by humans because they require human intelligence and conception.
Features	Features are the observable quantities and characteristics across all samples in the data set, either raw or mathematically transformed.
Feature selection	Feature selection is the process of selecting the most relevant features in developing a predictive model and can reduce the computational cost of modeling as well as improve the performance of the model.
Data augmentation	Data augmentation refers to techniques that can increase the diversity of training sets by applying random (but realistic) transformations, such as image rotation, flipping, scaling, etc.
Overfitting	Overfitting refers to a model that performs pretty well on the training data and fails to generalize and perform well in the case of unseen data scenarios.
Underfitting	Underfitting refers to a model that does not work correctly in the training data and also has poor performance in the new data.
Dimensionality reduction	Dimensionality reduction refers to techniques that reduce the number of random variables to the principal component of a data set.

In oncology studies, ML can analyze large-scale data in different format and combine them into predictions for tumor staging, cancer susceptibility, tumor recurrence, and patient survival (90). The process of ML is to extract knowledge from massive data sets, identify the underlying patterns, build predictive models, and finally make predictions on unseen data. A basic explanation of ML in cancer research can be achieved by considering the example of tumor recurrence prediction. Features from heterogeneous sources of data (clinical, imaging and genomic) are extracted by the ML algorithm. ML algorithm identify the combinations of specific features and tumor recurrence risk, and then build a prediction model. After that, when presented with a new case, the algorithm could provide the likelihood of recurrence for the new case.

### 4.1 Categories in machine learning

ML techniques can be generally categorized into three main groups based on whether the labels are required in the training data (91). Common categories of supervised and unsupervised learning can be found in **Table 3**.

**Table 3:** Categories of supervised learning and unsupervised learning for common algorithms.

Supervised Learning	Unsupervised Learning
Ordinary Least Square Regression	K-Means
Logistic Regression	Principal Component Analysis
Least Absolute Shrinkage Selection Operator Regression	Information Maximizing Component
Linear Discriminant Analysis	Self-organizing Maps
Ridge Regression	Topological Data Analysis
Elastic Net Regression	
Support Vector Machines	
Bayesian Networks	
Naïve Bayes Classifiers	
Random Forests	

#### 4.1.1 Supervised learning

The term ‘supervised’ refers to the technique where a model is supplied with labels, which are desired outcomes of the learning target (e.g., correct segmentation or classification results) (92). Generally, supervised learning is used to build a model to predict or categorize future events. It primarily focuses on classification (e.g., classifying benign or malignant tumors) and regression (calculating the risk of tumor relapse, estimating individualized disease-free survival, or predicting the length of patient life) (88).

#### 4.1.2 Unsupervised learning

Unsupervised learning is used when the input data has no labels. Hence, it learns the relationship between variables and uncovers patterns in unlabeled data. Supervised learning primarily addresses classification and regression issues, while unsupervised learning focuses more on dimensionality reduction and clustering (88). Clustering refers to identifying groups of similar cases within a data set based on some specific features; dimensionality reduction is used to reduce the complexity and heterogeneity of features extracted from massive biomedical data sets.

#### 4.1.3 Semi-supervised learning

Semi-supervised learning combines supervised and unsupervised ML. It can be helpful when only a tiny fraction of the data is labeled, or the labels on the input data are incomplete (93). A lack of sufficient labeled data frequently occurs in medical contexts because, given the complexity and variability of biomedical data, labeling information (e.g., correctly delineating the target in auto-segmentation) can be labor- and time-consuming. From this respective, semi-supervised learning can improve the efficiency and accuracy of information extraction for large data sets.

### 4.2 General workflow

#### 4.2.1 Data preparation

ML workflow usually starts with data acquisition and pre-processing. Data sets are typically split into training, validation, and evaluation sets. The predictive model is constructed on the basis of the training set and tuned by the validation set; finally, the model performance is assessed by the held-out evaluation set (89). In practice, the training set usually accounts for a larger fraction of the data (70%), whereas validation and evaluation sets usually make up 15%, respectively.

The prerequisites of ML success are a sufficient number of samples and high-quality data. To make the most of ML, enough training data size should be ensured to extract more generic features from the whole data set without over-emphasizing the impact from a few certain samples. Besides, the data quality should be checked to ensure input data’s appropriateness, reproducibility, and versatility. Specifically, for supervised learning, the correctness of the ground truth labels is also quite essential. Incorrect labels can significantly downgrade the model performance and are difficult to detect during training (86).

#### 4.2.2 Training and validation

The proper performance of the model relies heavily on features across sample sets, and model refinement can be achieved using the technique of feature selection. Inappropriate feature selection would undermine the training performance by straining computational resources, including time and memory. For ML application in TAMs, thousands of features can be used to predict the output variables (94), e.g., cell morphology, the molecular feature of TAMs, immune-related gene-based novel subtypes, patient characteristics, tumor infiltration, etc. After feature selection, ML would search for the optimal parameters and translate the features into accurate predictions. The parameters are created through a complicated calculation process.

After that, a validation set is also needed to optimize the parameters of the algorithm. In validation, a preliminary estimate of the model’s generalizability and accuracy is obtained; errors can be detected and corrected in this phase, and the process is then repeated (95). In other words, validation serves as a supplemental role in identifying the errors in a model in an early phase.

The input data is usually partitioned into k subsets of equal size. A single subset is retained as the validation set, and the remaining k-1 subsets are used as training data. The process of training and validation will continue until there is no further improvement in model performance.

#### 4.2.3 Evaluation

The evaluation data is used to assess the performance of the final model on samples outside the input data set (training and validation set). This process aims to estimate the model performance in the real-world. The evaluation set should be utilized at the very end of the research, avoiding the model being tuned to fit the evaluation set (96). The performance of a specific model relies on many factors, such as the data size and quality of training data, as mentioned above. The complexity and the relationship between the input and output variables, as well as the computational resources such as available training time and memory, all play essential roles in achieving high model performance (94).

## 5 ML algorithms used in TAMs

In this section, we are going to introduce the most common utilized ML algorithms applied in cancer research, especially, TAMs. We also compared the advantages and disadvantages of different algorithms in **Table 4** (97–101). Since the combination of ML and TAMs is an emerging cross-cutting research field, most studies were published in the last five years. All the matches were reviewed for suitability and significance for this review. **Table 5** depicts the publications we found most pertinent to our topic. Cancer type, sample size, research purpose, as well as the ML applications are presented in the table.

**Table 4:** Pros and cos of common machine learning algorithms.

	Pros	Cons
**Support Vector Machine**	Good performance with high dimensional data Good performance when classes are separable	Slow Cannot deal with overlapped classes Selecting appropriate hyperparameters is essential Selecting the appropriate kernel
**Principal Components Analysis**	Reduce overfitting Improve visualization Improve model performance	Independent variables become less interpretable Data standardization is necessary Lose information
**Naive Bayes**	Fast prediction Insensitive to irrelevant features Can be used for multi-class prediction Perform well with high dimensional data Less dependent to data size	Independence of features does not hold Relatively low prediction accuracy Zero Frequency
**Logistic Regression**	Simple to implement and interpret Feature scaling is unnecessary Perform well for linearly separable dataset Tuning of hyperparameters is unnecessary Fast at classifying unknown records	Assumption of linearity between the dependent variable and the independent variables Requires average or no multicollinearity between independent variables High reliance on proper presentation of data
**Random Forest**	Reduced error with high accuracy (balance the bias-variance well with multiple trees) Good performance on imbalanced datasets Can handle linear and non-linear relationships well Little impact of outliers Not prone to overfitting Useful for feature selection	Features need to have some predictive power Predictions of the trees need to be uncorrelated Not easily interpretable Computationally intensive for large datasets Black box nature
**Decision Tree**	Normalization or data scaling is unnecessary Can handle huge amount of data Easy to explain Easy visualization Automatic Feature selection Missing values does not affect building decision tree	Prone to overfitting A small change in data can cause large change in structure of decision tree Long training time Inadequate for applying regression and predicting continuous values
**K-Nearest Neighbor**	Simple to understand and implement No assumptions about data Constantly evolving model Can handle multi-class problem One hyper-parameter(k)	Slow Poor performance on datasets with large number of features Scaling is necessary Imbalanced data causes problems Outlier sensitivity No capability of dealing with missing values
**Artificial Neural Network**	High Efficiency High accuracy Multi-tasking Able to deal with incomplete information Having fault tolerance	Hardware dependence Black Box Nature Complex algorithm compared to traditional machine learning algorithms Need large data set

**Table 5:** ML algorithms and their applications in TAMs.

Authors and Years	Cancer Types	Sample Size	ML Algorithms	Research Purposes	ML Applications
Chang et al. (102) (2021)	Ovarian cancer	1566	Cox, LASSO	To construct macrophage related prognostic model for ovarian cancer	identify multiple features related to survival (uni-and multi-variate Cox) and construct the macrophage-related prognostic model (LASSO)
Rostam et al. (103) (2017)	/		Orange Data Mining Toolbox	To identify different macrophage functional phenotypes	auto-identification of phenotypes based on cell size and morphology (Orange)
Zhu et al. (104) (2019)	Rectal cancer	46	SVM	To investigate the role of tumor-infiltrating leukocyte cell composition in the prognosis of radiotherapy for rectal cancer	classify responsive and non-responsive patients (SVM)
Zhang et al (105) (2021)	Glioma	2405	NN, SVM ER, PCA	To investigate the predictive value of monocytes in the immune microenvironment and prognosis in glioma patients	validate clustering results (NN, SVM) and calculate the risk scores of patients (ER, PCA)
Zhang et al. (106) (2021)	Glioma	2365	Pamr, NN, SVM ER, PCA	To build a prognostic model based on the molecular feature of TAMs for gliomas	validate the clustering results (Pamr, SVM, and NN), construct risk scores (ER, PCA) and further validate the clustering results (SVM, NN)
Zhang et al. (107) (2020)	Prostate cancer	487	LASSO, PCA	To build a model to predict the risk of prostate cancer based on immune-related gene-based novel subtypes	determine the properties of the subtypes (PCA) and build the risk predictive model (LASSO)
Yin et al. (108) (2022)	Cervical squamous cell carcinoma	78	Cox, LASSO, LR, GMM	To investigate the roles of TAMs in the development of cervical squamous cell carcinoma	select immune‐related genes (Univariate Cox and LASSO), construct the risk score model (multi-variate Cox), build a diagnosis signature (LR), and then select the best models (GMM)
Yan et al. (109) (2020)	Ovarian cancer	365	Cox, LASSO, SVM, SVM-RFE	To explore prognostic genes associated with immune infiltration in ovarian cancer	identify the most valuable genes related to immune infiltration (LASSO, Cox), distinguish two different standards of immune infiltration (SVM), and work out the most valuable variables of immune infiltration (SVM-RFE)
Wu et al. (110) (2022)	Non-small cell lung cancer	681	RF	To develop a macrophages-based immune-related risk score model for relapse prediction in stage I–III non-small cell lung cancer	screen the robust prognostic markers and construct risk score to predict disease-free survival (RF)
Wei et al. (111) (2020)	Gastric cancer	407	SVM, LASSO, SVM-RFE	To investigate the effect of various components in gastric cancer TME and identify mechanisms exhibiting potential therapeutic targets	minimize the redundancy of features (LASSO) and rank the features (SVM, SVM-RFE)
Wang et al. (112) (2021)	Lung cancer	507	Mask R-CNN	To develop a prognostic model for the prediction of high- and low- risk lung adenocarcinoma	segment the nuclei of tumor, stroma, lymphocyte, macrophage, karyorrhexis and red blood cells (Mask R-CNN)
Vayrynen et al. (113) (2020)	Colorectal cancer	931	inform	To investigate the prognostic role of macrophage polarization in the colorectal cancer microenvironment	identify macrophages in tumor intraepithelial and stromal regions (inForm)
Ugai et al. (114) (2021)	Colorectal cancer	3092	inform	To investigate if the relationship between smoking and colorectal cancer incidence varies depending on macrophage infiltration	perform tissue category segmentation, cell segmentation, and cell type classification
Starosolski et al. (115) (2020)	Transgenic mouse models of neuroblastoma	16	Non-parametric neighborhood component analysis	To investigate if nanoradiomics can differentiate tumors based on TAM burden	radiomic features selection (the non-parametric neighborhood component method)
Shen et al. (116) (2021)	Brian tumor	3810	A self-developed deep learning algorithm based on contrastive learning	To stratify brain tumors for better clinical decision-making and prognosis prediction	distill expression signatures of transcriptome (DL)
Nakamura et al. (117) (2019)	Ovarian carcinoma	1656	SVM, RF, NN, LDA	To identify relationships between the expression of immune and inflammatory mediators and patient outcomes	classify ovarian cancer and normal tissue (SVM, RF, and NN) and map high-dimensional input data into a two-dimensional space (LDA)
Liang et al. (118) (2021)	Various cancers	9881	CART, LR, LDA, K-Neighbors Classifier, Gaussian Naive Bayes, SVM	To investigate the inflammasome signaling status to clarify its clinical and therapeutic significance	classify samples and validate gene set enrichment (all 6 ML methods)
Li et al. (119) (2021)	Bone-related malignancies	1675	RF	To investigate if a distinct immune infiltrative microenvironment exists in malignant bone-associated tumors and build a model for tumor diagnosis and prognosis	develop a bone-related tumor differential diagnosis model (RF)
Li et al. (120) (2022)	Gliomas	652	NN, LSTM, Cox, LASSO, RF	To predict survival and tumor-infiltrating macrophages in gliomas using MRI radiomics	extract significant radiomic features to construct a prediction model (NN, LSTM, Cox, LASSO, RF)
Kuang et al. (121) (2021)	Hodgkin lymphoma	130	LASSO, Cox, RF	To investigate potential markers for the diagnosis and prediction of classic Hodgkin lymphoma prognosis	identify prognostic genes and build a model for prognosis (LASSO, Cox, RF)
Hagos et al. (122) (2022)	Follicular lymphoma	32	ConCORDe-Net	To identify cell phenotypes and spatial distribution of immune cell subsets in the inter‐follicular area of follicular lymphoma TME	detect different immune cells within and outside neoplastic follicles (ConCORDe-Net)
Guo et al. (123) (2021)	Pulmonary sarcomatoid carcinoma	97	Cox, RF	To build an immune-based risk-stratification system for prognosis in pulmonary sarcomatoid carcinoma	construct a predictive model and rank the predictive ability of each variable (Cox, RF)
Lange et al. (124) (2018)	Uveal melanoma	64	HCA, PCA	To study the immune environment and explore whether absolute T-cell quantification and expression profiles can dissect disparate immune components	reveal cell-specific expression patterns in gene selection (HCA, PCA)
Lin et al. (125) (2022)	Adamantinomatous craniopharyngioma (ACP)	57	RF, LASSO	To study the molecular immune mechanism in ACP and find potential biomarkers for the targeted therapy for ACP	screen diagnostic markers (RF, LASSO)

Cox, Cox Proportional-hazards Regression; LSSO, Least Absolute Shrinkage and Selection Operator; PCA, Principal Component Analysis; ER, Elastic Regression; SVM, Support Vector Machine; Pamr, Prediction Analysis for Microarrays; LR, Logistic Regression; GMM, Gaussian Mixture Model; LDA, Linear Discriminant Analysis; SVM-RFE, Support Vector Machine Recursive Feature Elimination; NN, Neural Network; RF, Random Forest; CART, Classification and Regression Trees; ConCORDe-Net, Cell Count Regularized Convolutional Neural Networks; HCA, Hierarchical Cluster Analysis; LSTM, Long short-term memory; MLP, Multi-layer perceptron; Weka, Waikato Environment for Knowledge Analysis; ROF, Rudin-Osher-Fatemi.

### 5.1 Dimensionality reduction

**Figure 2:**
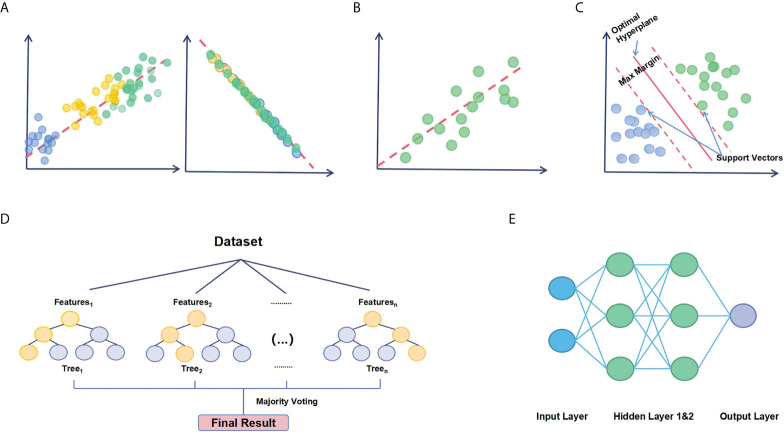
Basic principles of standard ML algorithms. **(A)** PCA reduces the dimensionality of a data set consisting of plenty of interrelated variables. **(A)** illustrates a series of data points viewed from another angle with approximately the same value on that dimension. It shows that the distinction between the data points can be represented by a principal component. **(B)** Regression analysis determines the relationship between factors and disease outcomes or identifies relevant prognostic factors for diseases. **(B)** illustrates regression estimating a mathematical formula that relates input variables to the output variable. **(C)** SVM generates a hyperplane in higher-dimensional feature space and maximizes the margin of error to select the best hyperplane. The best hyperplane would serve as a decision boundary for classification. **(D)** RF model ensembles a large number of small decision trees. Each tree is capable of making an individual prediction. **(E)** Neural networks tend to resemble the connections of neurons and synapses in human brain. The input data is assigned initial weights and transferred to output layers for classification. Hidden layers would tune the initial wrights to minimize the neural network’s prediction error.

Dimensionality reduction refers to techniques that transform data in high dimensions into a lower-dimensional form while preserving the relationships between the data points as much as possible. In a nutshell, it is a data preparation technique used for downsizing the input variables and performed before modeling. By far, Principal Component Analysis (PCA) is the most popular multidimensional data analysis technique (126). It reduces the dimensionality by eliminating less important components to omit the redundant dimensions and focusing only on the most important components that could best explain the heterogeneity in the data (**Figure 2A**). Other dimensionality reduction algorithms include t-distributed stochastic neighbor embedding and uniform manifold approximation and projection.

PCA is primarily applied to problems where there are a large number of features, which are referred to as high-dimensional problems (127). Generally, there are many important applications of PCA in cancer research because the input variables in oncology data are complex and massive. For example, PCA is used to extract principal components as signature score to calculate the patients’ risk scores based on meaningful macrophage-related genes (105, 106). Zhang et al. performed PCA on 487 patients to reduce the feature dimensions and clearly distinguished high-risk and low-risk patients (107). Autoencoder in deep learning neural networks is another method to perform dimensionality reduction. Encoder is the part of the model prior to the bottleneck. It aims to compress the data dimension to a bottleneck layer that is much smaller than the initial input data. Shen et al. developed a deep learning model through self-supervised feature representation learning to characterize immune infiltration from transcriptome (116). The developed model was used to distill expression signatures of the transcriptome in brain tumor samples. The application of PCA in TAMs research could potentially be promising in enhancing predictive accuracy when input variables and their inter-connections are remarkably complicated.

### 5.2 Regression

Regression analysis is a method to mathematically describe the relationships between the outcome of interest (e.g., patient survival or relapse risk) and one or more features, also termed as variables (**Figure 2B**) (128). It answers the questions: Which variable is the most significant? What’s the connection among these variables? And, perhaps most importantly, how certain are we about all of these variables? Regression analysis has been applied to cancer research for decades, from survival analysis with Cox’s proportional hazard regression to Least Absolute Shrinkage Selection Operator Regression (LASSO) regression for significant feature selection.

Linear regression is the most common and simplest model for discovering how one or more explanatory variables determine the dependent variable (129). Logistic regression is extended by a linear regression model for classification problems. However, it differs from linear regression by being employed when the outcome variable is binary. Yin et al. built a diagnosis signature by logistic regression based on selected significant factors correlated with TAMs. They found that these factors were conducive to distinguish normal tissues from tumor (108). Cox proportional hazard is generally used when the outcome is the time to an occurrence (for example, time to death, time to relapse). The results of Cox are explained in terms of a hazard ratio, indicating the risk of an event at a given time. Ridge regression and LASSO regression are variants of linear regression (linear regression appended with a regularization term) introduced for more accurate prediction. Ridge and LASSO are commonly used to reduce model complexity and prevent potential over-fitting. Typically, LASSO and Cox are combined together for disease prognosis. These studies generally use univariate Cox regression and LASSO regression to identify the significant characteristics and multivariate Cox regression to build risk score models (108).

Another variant of linear regression is elastic net regression. It integrates the LASSO and ridge regression methods by learning from their drawbacks to improve the regularization of statistical models. Thus, it achieves a more stable and better prediction than LASSO and ridge regression in less training samples. In two studies that intended to develop a prognostic model based on the molecular feature of TAMs, they both used elastic net to construct risk scores (105, 106). Especially, in Zhang et al’s study, they found that glioma with higher risk scores is populated by macrophages comprising both the traditional M1 and M2 phenotypes, which further indicates that M0/M1/M2 is a continuum rather than two extremes (106).

### 5.3 Classification

#### 5.3.1 Support vector machine

Support Vector Machine (SVM) is a powerful method that can be used for both regression and classification tasks (130). However, it mostly works as a classifier and aims to create a decision boundary, also termed as hyperplane, between two classes that distinctly classifies the data into different categories (131). The objective of SVM is to maximize the margin to select the best hyperplane, which offers some reinforcement so that subsequent data points can be classified with greater confidence. The margin is determined by a series of hyperplanes parallel to the decision boundary whose distance to the nearest data point is the largest in either the positive or negative class, as depicted in **Figure 2C**.

As a classifier, SVM is frequently used in TAMs. Patients can be classified into different groups based on the significant tumor-infiltrating immune cell proportions. For instance, patients with rectal cancer can be classified into responsive and non-responsive groups through the ML method based on the tumor-infiltrating immune cell composition and achieved an accuracy of 65% (104). Nakamura et al. applied SVM to discriminate between malignant and non-malignant tissues in ovarian cancer patients and malignant ovary samples through the immune signatures including M1 macrophage mediator signatures (117). Yan et al. used SVM to explore prognostic genes associated with immune infiltration and the classification accuracy reached as high as 0.934. Also of note, the high and low-risk groups exhibited significantly different proportions of TAMs (104). Some researchers used SVM to further validate the clustering results (105, 106). In an article by Liang, the authors applied six ML algorithms to predict inflammasome clusters, in which macrophages were the major immune cell population enriched in inflammasome complex^Mid^ and inflammasome complex^High^ clusters. In this paper, SVM achieved a highest prediction accuracy of 96% (118). Some researchers also use SVM-RFE, a feature selection algorithm that ranks the features according to the recursive feature deletion sequence, to identify prognostic genes associated with TAMs infiltration (109, 111).

The strength of SVM is that it can be used for complex data sets with many variables or dimensions. However, when it comes to high dimensions, SVM achieves a powerful model at the cost of easy interpretation of which features are influencing the model.

#### 5.3.2 Random forest

Random forest (RF) is an ensemble decision tree classifier combining multiple tree predictors introduced by Leo Breiman (132). As an ML algorithm near the top of the classifier hierarchy, the RF classifier is capable of ranking the predictive ability of each variable and constructing a predictive model (110). Generally, RF is based on the aggregation of a large number of uncorrelated and weak decision trees, and each uncorrelated tree casts an individual prediction. The final decision is made by majority voting of all trees, which outperform any single classifier (**Figure 2D**). RF models are considered less vulnerable to overfit the training data set given the large number of trees built, making each tree an independent model. Given a large number of trees ensembled and each tree indicating an independent model, random forest models are thought to be less susceptible to overfitting. The ability of RF to precisely classify observations is extremely valuable in oncology applications, such as predicting patient death or tumor relapse. So far, RF has been applied to many TAMs studies for classification. They are generally used to screen TAMs-related markers and construct an immune-related risk score for risk prediction (110, 121, 123, 125). By utilizing RFs, a diagnostic model based on immune infiltration can accurately perform the differential diagnosis of bone-related malignancies (119). Nakamura et al. used RF to investigate whether genes identified by literature search or other analysis can distinguish between normal tissues and cancer tissues (117). In many studies, RFs also worked with other algorithms to screen the overlapping markers, e.g., LASSO (121, 125).

Overall, the advantage of RF is that it is an ensemble algorithm which has more accuracy than any individual prediction, especially when multi-modality variables are combined (133). However, the high dimension of all the features in cancer research and their complex interactions make it very difficult for humans to interpret the model and results.

### 5.4 Neural networks and deep learning

Deep learning (DL) is a notable sub-class of ML which has a remarkable ability to learn patterns from raw, unstructured input data by incorporating artificial neural networks (ANN) (134). ANN is inspired by the structure and function of the brain. It attempts to use multiple layers of calculation units to imitate how the human brain processes input information. It is essentially a mathematical model consisting of an input layer, multiple hidden layers, and an output layer, as shown in **Figure 2E**. Each layer has multiple artificial neurons, also known as nodes in neural network. The nodes in input layers gather source material such as image pixels and numerical data. Hidden layers in the middle connect nodes to the next layer, creating non-linear representations between source data and the output layer (135).

Despite deriving from ANN, the DL framework differs from a straightforward neural network. Overall, DL networks are larger and consist of more layers and nodes, making it possible to reflect complicated interrelationships precisely. DL is able to process plenty of features across a large number of samples and derive neural network-based ‘representations’ quickly. Many specialized DL models have outperformed traditional ML models for various tasks, such as medical image segmentation and image-based tumor staging. Classical DL algorithms include Convolutional Neural Network (136), Recurrent Neural Networks (137), Radial Basis Function Networks (138), Long Short-Term Memory Networks (LSTMs) (139), Self-Organizing Maps (140), Autoencoders (141), etc., which have been proved to achieve state-of-the-art performance in specific applications (142–144).

Applications of neural networks and DL in TAMs focus more on classification and medical image segmentation. Li et al. developed an MRI radiomics approach to predict survival and tumor-infiltrating macrophages in gliomas (120). They used two neural network models and one long short-term memory DL model to divide patients into long and short-term survival clusters. In research conducted by Wang et al. (112), Mask R-CNN, a DL-based model, was applied to segment the nuclei of the tumor, lymphocyte, stroma, karyorrhexis, red blood cells and macrophage from pathology images. In addition to the existing segmentation algorithms, some studies developed their own DL segmentation models to characterize immune infiltration. Risom et al. segmented cell nuclei using Msmer, a DL-based algorithm developed in their lab (145), and Hagos et al. used ConCORDe-Net to detect cells in multiplex immunohistochemistry images (122). Meanwhile, commercial and Open-source software could also be used for segmentation in cancer research. For example, inForm software package (Akoya Biosciences) has been applied in some studies to automatically perform tissue category segmentation, cell segmentation, and cell type classification (113, 114). InForm software is a powerful software that enables per-cell analysis of immunohistochemistry and immunofluorescence. It allows the separation and measurement of weak and spectrally overlapping markers and automatic detection and segmentation of specific tissues. Orange Data Mining Toolbox is another open-source software. Rostam et al. used it to automatically identify different macrophage functional phenotypes based on cell size and morphology (103).

Interest in DL models has grown in recent decades owing to rapid advances in high-performance computing infrastructure, such as cloud and GPU computing (146). However, it is still far from meeting the vast amounts of data needed for medical research. Developing deep neural networks and then training is time-consuming and computationally expensive compared with traditional ML methods.

## 6. Challenges

Despite such exciting research, various limitations or requirements must be addressed before ML can realize its full potential in the studies focusing on TAMs. As most ML models are data-driven, the most critical challenge is the requirement of tremendous and valuable data sets (147). Generally, data related to TAMs can be incredibly complex, with thousands of variables capturing different facets of the TME system. However, these data sets are still too small for ML modeling, especially for unsupervised learning. The lack of sample size might lead to poor model performance or overfitting. Deep neural networks are especially vulnerable to overfitting because they have thousands to millions of parameters.

Moreover, data quality and completeness are also challenging in the studies of tumor prognosis, in which patient follow-up might be irregularly collected or lost, and different institutions may use various standards of testing. In response to the challenge of massive clinical data acquisition, some cloud-based cancer repositories such as Gene Expression Omnibus (GEO) and The Cancer Genome Atlas (TCGA) have been created to enable cross-institution data sharing and data quality assurance. We hope with the emergence of more open-source data sets and data standardization, these restrictions will be less of an issue in the future.

Clinical translation is also a challenge for ML. Many trials are still in the stage of by-proof-test. Research groups and companies are facing the challenges of making their products more reliable and practical in large-scale implementations or even real usage scenarios. Similarly, many innovative solutions, generated from the frontiers of ML research and shown to be theoretically powerful, have yet to integrate into day-to-day clinical use. In modeling, most models take fixed training and testing data set, which is impractical in real clinical practice. Considering the rapid changes in tumor data, continuous updating and reevaluation are required to monitor the model performance and guarantee model consistency. In addition, most of the current ML-based tumor models are single-center studies. There are considerably fewer external validation studies of TAMs in the published papers. Future studies should involve external or cross-institution validation to ensure the test set is diversified enough with different clinic scenarios involved. We believe the robust external validation and improvements in interpretability and generalizability may boost clinician confidence in ML and facilitate further incorporation of ML models into clinical practice.

Furthermore, after reviewing papers combining ML with TAMs, we come to realize that the complexity and heterogeneity of TAMs in TME are far from being fully elucidated. As discussed above, the dichotomy of TAMs is too simple to clarify macrophage activation states *in vivo*. What should be noted is that M0/M1/M2 is a continuum *in vivo* instead of well-delineated categories. TAMs are characterized by its remarkable plasticity. The phenotypes can switch between the two extremes, while most existing studies still regard TAMs as two distinct extremes. Besides, subtypes of M2-TAMs can be further identified and classified as M2a, M2b, M2c (148, 149), and M2d in TME. Identifying complexity and heterogeneity of TAMs *in vivo* and the subtypes of M2 macrophages more precisely to reduce side effects of cancer therapy using ML methods can be challenging but promising. Therapies addressing the recruitment, depletion and repolarization of M2 are promising strategies for tumor treatment. With the help of ML, many studies are enabled to identify specific molecules involved in polarization of M0 macrophages towards M1/M2 macrophages and TAMs recruitment. However, the key biomarkers in depletion and repolarization of M2 based on ML have not received a lot of attention. By integrating more medical images and omics data, it is anticipated that ML will have broader prospects on exploring, validating and implementing critical genes in the repolarization of TAMs to further facilitate precision oncology.

## 7. Future directions

ML in cancer research is still in the early stage of exploration. More investigations and efforts are required to break through current limitations. In terms of reducing the need for a large data set, Generative Adversarial Networks (GAN) are receiving attention. GAN has two neural networks, which are generative and discriminator networks. They contest with each other in a zero-sum game and generate new and synthetic instances of data that can ‘fool’ the discriminator network.

Precision medicine is the future direction of cancer therapy, in which case patients can get optimized management and treatment to improve survival. An important part of precision oncology involves understanding cancer genomics, radiomics and the complex heterogeneity of TME. With the help of ML, scientists are able to disentangle more cancer characteristics, enabling precision oncology. One of the popular and evolutionary directions in ML is reinforcement learning. It learns to achieve goals in an uncertain and complex environment. Due to the non-stationary tumor environment with changing conditions and stimuli, reinforcement learning has the potential to offer computer-guided decision support for personalized treatment. Currently, its applications in medicine are mainly focus on medical image analysis, disease screening and personalized treatment recommendations. In the future, we envision that it could be employed for dynamic cancer treatment regimens after personalized tumor prognosis, tailoring the treatment for each individual.

Overall, the combination of ML and TAMs is relatively young and far from fulfilling its potential in cancer research. The distinctive nature of cancer studies makes accuracy and interpretability extremely crucial. We still have a long way to go to uncover and harness the intricacies of ML and the complexities of TME. Hopefully, with ever-evolving algorithms, more potent supercomputers, and substantial investment being involved in this field, these applications will be more intelligent, cost-effective, and time-efficient. In the future, ML is expected to play a more critical role in TAMs analysis and precision oncology.

## Author contributions

ZL and QY wrote the manuscript; QZ and XY reviewed and edited the paper; ZBL and JF offered technological guidance. All authors reviewed the results and approved the final version of the manuscript. ZL and QY are the co-first authors and JF is the corresponding author.

## Funding

This study was supported by the Science and Technology Project of Shanghai Municipal Science and Technology Commission (No.22Y31900500).

## Conflict of interest

The authors declare that the research was conducted in the absence of any commercial or financial relationships that could be construed as a potential conflict of interest.

## Publisher’s note

All claims expressed in this article are solely those of the authors and do not necessarily represent those of their affiliated organizations, or those of the publisher, the editors and the reviewers. Any product that may be evaluated in this article, or claim that may be made by its manufacturer, is not guaranteed or endorsed by the publisher.
